# Factors Associated with Parents’ Perceptions of Parental Smoking in the Presence of Children and Its Consequences on Children

**DOI:** 10.3390/ijerph10010192

**Published:** 2013-01-07

**Authors:** Yu-Ting Chen, Fei-Hsiu Hsiao, Nae-Fang Miao, Ping-Ling Chen

**Affiliations:** 1 Graduate Institute of Nursing, College of Nursing, Taipei Medical University, No. 250, Wu-Hsing Street, Taipei City 110, Taiwan; E-Mail: annie@mail.cgu.edu.tw; 2 School of Nursing, College of Medicine, Chang-Gung University, No. 259, Wen-Hwa 1st Road, Kwei-Shan, Tao-Yuan 333, Taiwan; 3 Department of Nursing, College of Medicine, National Taiwan University, No. 1, Sec. 4, Roosevelt Road, Taipei, 10617, Taiwan; E-Mail: hsiaofei@ntu.edu.tw; 4 School of Nursing, College of Nursing, Taipei Medical University, No. 250, Wu-Hsing Street, Taipei City 110, Taiwan; E-Mail: naefang@tmu.edu.tw; 5 Graduate Institute of Injury Prevention and Control, College of Public Health and Nutrition, Taipei Medical University, No. 250, Wu-Hsing Street, Taipei City 110, Taiwan

**Keywords:** parental smoking, perception, secondhand smoke, children

## Abstract

Parental smoking is the major source of children’s secondhand smoke exposure and is influenced by parents’ perception of children’s exposure. However, the factors associated with these perceptions remain unclear. The objective of this study was to examine factors associated with parents’ perceptions about parental smoking in the presence of children and its consequences. We conducted a cross-sectional study on parents’ perceptions of parental smoking and measured their evaluations of its consequences using a self-report questionnaire. Other variables include socio-demographic characteristics and smoking-related experience. Results show that parents’ gender, education level, occupational type, smoking status, and agreement on a home smoking ban independently predict parents’ evaluation of the consequences of parental smoking in the presence of children. Parents’ gender, education level, annual family income, smoking status, agreement on a home smoking ban, and evaluation of the consequences of parental smoking independently predicted parents’ perceptions. Findings indicated that a specific group expressed greater acceptance of parental smoking and was less aware of its risks. Motivating parents to create a smoke-free home and increasing awareness of the adverse consequences of parental smoking is beneficial in reinforcing attitudes opposed to parental smoking.

## 1. Introduction

Secondhand smoke (SHS) causes serious harm to children’s health, including respiratory symptoms, middle ear infections, allergies, asthma, decreased in lung function and cognitive function, and increased emotional arousal and behavioral problems [1,2,3,4,5,6]. Most adults are aware of the adverse health of SHS [7,8] and support smoking bans in public areas. However, approximately 40% of children are still exposed to SHS at home [9]. In the United States, parental smoking is the main source of 25%–43% of children’s exposure to SHS in homes [10,11]. A survey from Hong Kong reported that 34% of school-aged children live with at least one smoking parent [12]. In Taiwan, approximately 45% of junior high and 47% of senior high school students are exposed to SHS at home, with half of them exposed to SHS at home on a daily basis [13,14]. This SHS exposure at home among adolescents is higher than in other countries.

Parents’ attitudes toward children’s exposure to SHS and awareness of its risks are associated with children’s exposure to SHS [15,16,17]. However, some adults allow children to be exposed to household smoking, even when they are aware of its harmful effects. This might be because some studies, instead of emphasizing parental smoking as a source of SHS, do not directly highlight the source of exposure when measuring attitudes toward, or risk awareness of, children’s SHS exposure. Parental smoking is a major source of children’s exposure to SHS [4]. Fewer than 20% of parental smokers completely restrict smoking at home [18,19], resulting in children’s exposure. Thus, focusing on parental smoking in the vicinity of children may be better than focusing on children’s exposure to SHS when measuring parents’ perceptions of it. This is particularly true when parents are expected to be the main people striving to protect children from SHS exposure. Therefore, identifying the factors that influence parents’ perceptions toward parental smoking is important. These factors could then serve as a reference in the design of interventions to effectively improve parents’ attitudes against parental smoking.

Qualitative studies have explored parental perceptions about household smoking and children’s exposure to it. These perceptions include viewing smoking as a habit or as a cultural norm, respecting household smokers’ and friends’ right to smoke, valuing hospitality toward visitors who smoke [20,21,22], and the belief that children from smoking families are at no greater health risk than children from non-smoking families [23]. Previous studies focused on exploring perceptions rather than examining the factors associated with these perceptions, and the sample sizes were relatively small (fewer than 54).

Carlsson *et al.* [24] identified that parents’ education level and smoking status are related to their attitudes toward, and risk awareness of, parental smoking. However, only one question in each actually measured attitudes and risk awareness, and another question related to attitude was directly targeted at smokers who might induce misinterpretation for nonsmokers to answer. Additional findings on parental characteristics associated with their perceptions regarding parental smoking require quantitative studies and larger samples. According to the integrative model of behavioral prediction (the latest formulation of the Theory of Reasoned Action and Theory of Planned Behavior), an individual’s attitudes and beliefs are influenced by personal background factors, such as socio-demographic characteristics and behavioral patterns [25]. The family pattern is also related to parents’ attitude toward smoking [16]. The adoption of a home smoking ban reduces children’s exposure to household smoking [19,26]. This might suggests that parents’ attitudes toward a home smoking ban are associated with the perception of parental smoking and its consequences. Therefore, we conducted this study to determine the factors associated with parents’ perceptions about parental smoking in the presence of children and their evaluation of its consequences. These factors include socio-demographic characteristics, parents’ smoking status, and agreement on a home smoking ban. The purpose of this study are as follows: (a) to identify the sociodemographic predictors of parents’ perceptions of parental smoking and their evaluation of its consequences; (b) to examine if smoking status and agreement on a home smoking restriction, combined with the sociodemographic predictors described, are associated with parents’ evaluation of the consequences of parental smoking in the presence of children; and (c) to examine if parents’ evaluation of the consequences of parental smoking, combined with the variables described, is associated with parents’ perception regarding parental smoking in the presence of children.

## 2. Experimental Section

### 2.1. Study Design and Sample

We conducted a cross-sectional study to collect data from the parents of Taiwanese school-aged children in April and May of, 2010. Counties and cities in Taiwan were classified in five geographic locations (northern, central, southern, eastern, and off-island regions) according to their level of development and access to resources. Four cities/counties—one in each area (northern, central, southern, and eastern regions)—were designated for sample selection to achieve the optimal representativeness of the sample. Off-island regions were excluded because only 0.4% of the Taiwanese population resides there. We contacted elementary schools selected conveniently from these four counties/cities and invited the parents of school-aged children to participate in the study. Five schools agreed to participate: two schools in Northern Taiwan, and one school each in Central, Southern, and Eastern Taiwan. Two classes were randomly selected from each grade (from first to sixth) at each participating school. To ensure equal gender representation, each selected paired class was randomly assigned in a cluster to invite either the father or mother of each student to participate in the study. Based on this selection process, 62 classes from five schools were incorporated into the study. Selected parents were sent an invitation letter with an introduction to the study, consent form, and self-administered anonymous questionnaire, all of which were voluntarily returned to researchers in a sealed envelope. Because the target participants were parents of school-aged children, the questionnaires completed by caregivers other than parents were excluded. All collected envelopes were then mailed to the researchers. A small gift was given to each parent and teacher in recognition of the time they had contributed. This study was approved by the Institutional Review Board of a university for the protection of human subjects (IRB No.: P960233).

The questionnaire filled out by participants was administered in Chinese, and included the following parts: (a) perceptions of parents smoking in the presence of children (PSPC); (b) consequence evaluations of parents smoking in the presence of children (CESPC); (c) smoking status and experience of quitting smoking; (d) experience with smoking restrictions at home; and (e) demographic information. The development of scale PSPC and scale CESPC, in the Chinese version, were based on results from a preliminary qualitative study exploring parents’ experience and perceptions about parental smoking in the presence of children. The content of both scales was revised to improve relevancy and clarity in accordance with the CVI criteria suggested by Lynn [27], the suggestions of experts (specializing in public health, community health nursing, or tobacco control and policy), and the discussions of the research team. These scales were also tested for validity and reliability with a sample of parental smokers living with school-aged children. Construct validity was established using factor analysis, resulting in a three-subscale structure consisting of the scale PSPC and a two-subscale structure of scale CESPC. A test of internal consistency for two scales and subscales of the revised version showed satisfactory reliability. The detailed development and psychometric testing of the scales are under review elsewhere.

### 2.2. Measurements

#### 2.2.1. Perceptions of Parents Smoking in the Presence of Children (PSPC)

The 8-item scale adopted in this study assessed how parents perceived parental smoking in the presence of their children. This scale contained three subscales: “adverse behavior” included 3 items to assess the negative aspects of smoking in the presence of children; “harmless behavior” included 3 items that reflected an acceptance of smoking behavior; and “rational needs” included 2 items that valued the need for smoking. All items were scored on a 5-point Likert-type scale, ranging from 1 (*strongly agree*) to 5 (*strongly disagree*). The mean scores for scales and subscales were calculated, with a higher score indicating greater agreement with the perception. To calculate the mean score of the PSPC scale, the scores of each item from the subscales “harmless behavior” and “rational needs” were reversed as 5 (*strongly disagree*) to 1 (*strongly agree*). A higher score on the PSPC scale indicated a more negative perception of parents smoking in the presence of their children. Conversely, a score of each item was not reversed when calculating the mean score of the subscales “harmless behavior” and “rational needs”. In other words, a higher score on the “harmless behavior” and “rational needs” subscales indicated greater acceptance of viewing parents smoking in the presence of their children as harmless and rational needs. Cronbach’s alpha values for the scale and subscales of PSPC ranged from 0.63 to 0.74.

#### 2.2.2. Consequence Evaluation of Parents Smoking in the Presence of Children (CESPC)

This study measures parents’ evaluation of the consequences of parental smoking in the presence of children using the CESPC scale. This 6-item scale contains two subscales with three items in each: “physical/environmental aspect” determines the effect on children’s health and the environment, whereas “psychosocial aspect” measures the effect on children’s behavior, emotions, and interpersonal relationships. All items were scored on a 5-point Likert-type scale, ranging from 1 (*strongly agree*) to 5 (*strongly disagree*). The mean scores for each subscale and total items were calculated, with a higher score indicating greater agreement with negative effects caused by parental smoking. Cronbach’s alpha values for the scale and subscales of CESPC ranged from 0.70 to 0.88.

#### 2.2.3. Home Smoking Restrictions

This study uses two questions to measure parents’ perspectives (*i.e.*, *agree or not*) and adoption of home smoking restrictions: “Do you agree with smoking restrictions at home?” and “Has your family adopted a home smoking ban?”

#### 2.2.4. Smoking Status

Smoking status was measured with two questions: “Have you smoked more than 100 cigarettes in your lifetime?” and “Approximately how many days have you smoked during the past 30 days?” Parents who reported smoking more than 100 cigarettes in the past and who had smoked at least one day in the past 30 days were defined as current smokers. In contrast, parents who reported never having smoked or smoked fewer than 100 cigarettes in the past were defined as nonsmokers.

### 2.3. Statistical Analysis

To select variables for possible associated factors, we compared the differences in parents’ perceptions and evaluation of consequences between various demographic characteristics, smoking status, and agreement on home smoking restrictions at the bivariate level by using independent *t* tests and ANOVA. Significant variables in the bivariate test were selected for a regression model to examine the predictors of parents’ perceptions and evaluation of the consequences of parental smoking. We used hierarchical linear regression to choose the best set of variables to explain the total variance of parents’ perceptions about parental smoking in the presence of their children, and also their evaluation of its consequences. A partial F test was then performed to determine the model that best fit in predicting outcome variables.

## 3. Results

### 3.1. Participant Characteristics and Experience with Home Smoking Restrictions

We invited 1,670 parents to participate in this study. Of these, 1,520 responded to the study (response rate: 91%). Fifty-four parents were excluded from data analysis because they either provided unidentified information or failed to answer two major scales (perceptions about parents smoking in the presence of children and evaluations of the consequences for children). Overall, 1,466 parents (53.2% mothers and 46.8% fathers) completed the questionnaire and were included in the analysis. Thirty-seven percent of parents lived in Northern Taiwan, and approximately 24%, 17.6%, and 21.4% were from the central, southern, and eastern regions of Taiwan, respectively. Table 1 shows the demographic characteristics of participants. Most parents were 30–39 years (47.3%) and 40–49 years of age (45.8%). Forty-seven percent of parents have a college education level, followed by an education level of senior high school (41.4%). Most participants (86.8%) lived with their spouse and children. Almost 49% of parents worked in the service field. Approximately half (50.17%) reported an annual family income of less than NT$600,000 (US$18,750), representing the lowest 40%–50% of the 2010 average disposable income in Taiwan [28].

In responding to questions related to home smoking restrictions, nearly 85% of all parents agreed with smoking restrictions in the home. However, among the 819 parents who either smoked or lived with smokers, only 43.7% banned smoking in the home.

### 3.2. Perceptions and Evaluation of the Consequences of Parental Smoking in the Presence of Children

The mean scores on the PSPC scale and subscales—adverse behavior, harmless behavior, and rational needs—were 4.75, 4.42, 1.95, and 2.41, respectively (Table 1). These results show that these parents agreed that smoking in the presence of children was an adverse behavior, disagreed with viewing it as harmless, and fairly disagreed that smoking entailed rational needs. These parents also agreed that parental smoking in the presence of children had a physical/environmental and psychosocial effect (mean score: 4.59, 3.94) on children (Table 2).

### 3.3. Determinants of Perceptions about Parental Smoking in the Presence of Children, and Their Evaluation of its Consequences

The results of the bivariate test show a significant difference in parents’ perceptions of parental smoking in the presence of children between different gender, education level, family type, type of occupation, level of annual family income, location of children’s school, parents’ smoking status and their status of agreement on smoking restriction at home (Table 1). A bivariate test identified the same variables as significant when comparing the difference in parents’ evaluation of the consequence (Table 2). Therefore, we included these variables in the regression model. The regression model also included parents’ evaluation of consequence to determine parents’ perception of parental smoking since we hypothesize that an individual’s perception about a behavior is influenced by the outcome of that behavior. Hierarchical multiple regression was applied separately to examine determinants of evaluation of consequences and the determinants of perceptions. All dependent variables were coded as dummy variables. In analyzing the factors determining parents’ evaluation of consequences, the variable of smoking status was entered first. Results indicate that parents’ smoking status predicted their evaluation of consequence from parental smoking in the presence of children (adjusted R^2^ = 11.21%, F = 182.75, *p* < 0.001). We then entered the variable of parents’ agreement with having home smoking restrictions. Results show an increased adjusted R^2^ to 13.65% in Model 2 (partial F = 88.65%, *p* < 0.001), indicating an improvement of fit of the regression model compared with Model 1. Finally, significant demographic variables (gender, educational level, occupational type, family type, annual family income, and children’s school location) were entered in the last step. The final model with all the variables entered revealed a significant increase of 3.58% in the adjusted R^2^ compared with Model 2 (partial F = 12.37, *p* < 0.001). This model explained 17.23% of the variance in participants’ awareness of the risk of parents smoking in the presence of their children. In this model, the variables of parents who agreed with having smoking restrictions in the home (β = 0.25, *p* < 0.0001), were mothers (β = 0.07, *p* = 0.008), and an education level of senior high school (β = 0.18, *p* = 0.0008) or higher (β = 0.25, *p* < 0.0001) was independently associated with more agreement on the negative consequence of parental smoking in the presence of children (Table 3). In addition, parents who smoked (β = −0.42, *p* < 0.0001) or worked in the agricultural/industrial field (β = −1.05, *p* = 0.004) were less likely to agree with the negative consequences of parental smoking in the presence of children.

To analyze the factors determining parents’ perceptions, their evaluation of consequence (mean score of the CESPC scale) was entered in Step 1, smoking status was entered in Step 2, agreement with having home smoking restrictions was added in Step 3, and demographic variables such as gender, education level, occupational type, family type, annual family income, and children’s school location) were added in the final step. Table 4 shows the results of hierarchical regression analysis. In Model 1, parents’ evaluation of consequence was a significant predictor of perceptions (adjusted R^2^ = 36.93%, F = 858.7, *p* < 0.001). Adding the variable of smoking status produced a significant increase of 0.75% in the adjusted R^2^ that emerged (adjusted R^2^ = 37.68%, partial F = 54.22%, *p* < 0.001). Adding the variable of agreement with having home smoking restrictions in Model 3 increased the adjusted R^2^ by 1.73% (adjusted R^2^ = 39.41%, partial F = 67.39, *p* < 0.001). Entering all the variables in Model 4 improved the fit with the regression model compared with Model 3 (partial F = 11.97, *p* < 0.001), with an increase of 2.21% in the adjusted R^2^. Model 4 explained 41.62% of the variance in participants’ perceptions of parents smoking in the presence of their children. In this model, the variables of parents who were aware of a greater risk from parental smoking (β = 0.54, *p* < 0.0001), agreed with having smoking restrictions in the home (β = 0.16, *p* < 0.0001), were mothers (β = 0.16, *p* < 0.0001), with a college education level or above (β = 0.11, *p* = 0.03), and an annual family income of more than NT$1,000,000 (β = 0.11, *p* = 0.03) were independently associated with their opposition of parents smoking in the presence of their children. In addition, parents who smoked were more likely to accept parental smoking in the presence of children (β = −0.08, *p* = 0.04).

## 4. Discussion

This study identified parents’ gender, education level, and certain occupation types as sociodemographic predictors of parents’ evaluation of the consequences of parental smoking in the presence of children. This study also shows that parents’ gender, education level, and annual family income are sociodemographic predictors of their perceptions toward parental smoking in the presence of children. Mothers with a higher education level expressed more perceptions opposed to parental smoking, and more agreement with the adverse consequences of exposing children to parental smoking. Women usually assume more responsibility for taking care of their children than do men [29]. Therefore, women pay more attention to information related to children’s health. Parents’ education level had a trend effect on their perceptions toward parental smoking around children and their evaluations of its consequence. Specifically, the degree of the perception opposed to parental smoking and agreement with its negative consequence increased as the level of education increased. The predictors of education level, occupational field, or annual family income may reflect a group of blue-collar workers who work harder to earn money and make a living.

**Table 1:** Comparison of perceptions of parental smoking in the presence of their children between different sociodemographic characteristics, smoking status, and agreement with having home smoking restriction (N = 1,466).

Variables	Distribution of Characteristics		Perceptions-Overall			Perceptions-Adverse behavior			Perceptions-harmless behavior			Perceptions-rational needs		
	n	%	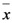	SE	f/t ^a^	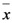	SE	f/t ^a^	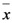	SE	f/t	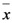	SE	f/t ^a^
**Total participants**	1,466		4.75	0.65 ^b^		4.42	0.66 ^b^		1.95	0.97 ^b^		2.41	0.98 ^b^	
**Gender**					−9.12 ***			−8.30 ***			4.72 ***			8.91 ***
Male	679	46.76	3.92	0.03		4.27	0.03		2.07	0.04		2.65	0.04	
Female	773	53.24	4.22	0.02		4.55	0.02		1.83	0.03		2.20	0.03	
*Age*					1.26			0.54			2.34			0.78
① 29 or under	43	2.97	3.91	0.09		4.35	0.09		2.21	0.15		2.58	0.12	
② 30–39	685	47.34	4.08	0.02		4.44	0.02		1.94	0.04		2.41	0.04	
③ 40–49	662	45.75	4.08	0.02		4.41	0.03		1.91	0.04		2.42	0.04	
④ 50 or above	57	3.94	4.01	0.11		4.37	0.10		2.14	0.14		2.30	0.15	
**Marital status**					0.37			0.07			−1.05			0.41
Married	1,258	86.76	4.08	0.02		4.42	0.02		1.93	0.03		2.42	0.03	
Other	192	13.24	4.06	0.05		4.42	0.05		2.01	0.07		2.38	0.07	
**Education Level**					39.36 ***			25.24 ***			45.48 ***			3.75 *
① Junior high or under	162	11.15	3.78	0.05		4.15	0.06		2.39	0.08		2.57	0.07	
② Senior high	601	41.36	4.00	0.03	③ > ② > ①	4.37	0.03	③ > ② > ①	2.08	0.04	③ < ② < ①	2.44	0.04	
③ College or above	690	47.49	4.22	0.02		4.53	0.02		1.71	0.03		2.35	0.04	③ < ①
**Family type**					3.54 ***			2.98 **			−2.67 **			−2.47 *
Nuclear family ^c^	856	58.55	4.13	0.02		4.46	0.02		1.89	0.03		2.36	0.03	
Others	606	41.45	4.00	0.03		4.36	0.03		2.03	0.04		2.49	0.04	
**Occupation**					7.85 ***			5.65 *			7.01 ***			3.29 *
① Agriculture/industry	313	21.78	3.96	0.04	① < ③	4.30	0.04	① < ③	2.04	0.05	① > ③	0.57	0.04	① > ③
② Business	154	10.72	4.04	0.05	② < ③	4.43	0.05		2.03	0.08	② > ③	0.46	0.07	
③ Service-public, social & health related	332	23.10	4.23	0.03		4.54	0.03		1.71	0.05		2.31	0.05	
④ Service-others	369	25.68	4.08	0.03	④ < ③	4.40	0.04		1.92	0.05		2.40	0.05	
⑤ Unemployed	269	18.72	4.06	0.04	⑤ < ③	4.45	0.04		2.04	0.06	⑤ > ③	2.35	0.06	
**Annual family income**					13.57 ***			6.79 ***			16.33 ***			4.12 **
① 300,0000 or under	292	20.43	3.90	0.04		4.32	0.04	① < ⑤	2.27	0.06	① > ② > ⑤	2.51	0.06	
② 300,001–600,000	425	29.74	4.05	0.03	⑤ > ② > ①	4.37	0.03	② < ⑤	1.98	0.04	② > ④	2.39	0.05	
③ 600,001–800,000	262	18.33	4.07	0.04	⑤ > ③ > ①	4.36	0.04		1.87	0.06	① > ③	2.56	0.06	③ > ⑤
④ 800,000–1,000,000	180	12.60	4.21	0.04	④ > ①	4.49	0.05		1.72	0.06	① > ④	2.32	0.07	
⑤ 1,000,001 or above	270	18.89	4.26	0.04		4.57	0.03		1.70	0.05		2.27	0.06	
**Children’s School location**					6.80 ***			6.60 ***			4.89 **			1.78
① North	544	37.11	4.11	0.03	① > ③	4.46	0.02	① > ③	1.90	0.04	① < ③	2.39	0.04	
② Central	350	23.87	4.11	0.03	② > ③	4.48	0.03	② > ③	1.94	0.05		2.39	0.05	
③ South	258	17.60	3.91	0.04		4.26	0.04		2.15	0.06		2.54	0.05	
④ East	314	21.42	4.11	0.04	④ > ③	4.41	0.04		1.87	0.05	④ < ③	2.38	0.06	
**Children’s grade**					0.18			0.68			0.46			0.43
① Grades 1–2	478	32.61	4.09	0.03		4.44	0.03		1.92	0.04		2.44	0.04	
② Grades 3–4	487	33.22	4.08	0.03		4.42	0.03		1.94	0.04		2.42	0.05	
③ Grades 5–6	501	34.17	4.06	0.03		4.39	0.03		1.98	0.04		2.38	0.04	
**Smoking status**					−12.62 ***			−9.97 ***			7.50 ***			11.16 ***
Smoker	336	23.32	3.71	0.03		4.11	0.04		2.28	0.05		2.92	0.05	
Non-smoker	1,105	76.68	4.19	002		4.52	0.02		1.84	0.03		2.27	0.03	
**Agree with smoking restrictions at home**					7.39 ***			5.93 ***			−6.63 ***			−4.61 ***
Yes	1,217	84.69	4.14	0.02		4.48	0.02		1.87	0.03		2.37	0.03	
No	220	15.31	3.75	0.05		4.14	0.05		2.33	0.07		2.70	0.07	

^a^ f value by one-way ANOVA test for comparison of three and more variables, t value by independent t test for comparison of two variables; ^b^ standard deviation; ^c^ living with spouse and children only; * *p* < 0.05, ** *p* < 0.01, *** *p* < 0.001.

**Table 2:** Comparison of evaluation of the consequences of parental smoking in the presence of the children between different sociodemographic characteristics, smoking status, and agreement with having home smoking restriction (N = 1466).

Variables	Risk awareness-Overall				Risk awareness—Psycho-social			Risk awareness—Physical/environmental		
	n	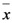	SE	f/t ^a^	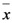	SE	f/t ^a^	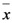	SE	f/t ^a^
**Total participants**	1,466	4.27	0.66 ^b^		4.59	0.59 ^b^		3.94	0.87 ^b^	
**Gender**				−7.04 ***			−5.73 ***			−7.21 ***
Male	679	4.13	0.03		3.80	0.03		4.47	0.02	
Female	773	4.38	0.02		4.06	0.03		4.70	0.02	
*Age (mean 39.54, sd = 5.5)*				1.26			1.08			1.57
① 29 or under	43	4.15	0.10		3.79	0.13		4.52	0.09	
② 30–39	685	4.26	0.02		3.91	0.03		4.60	0.02	
③ 40–49	662	4.29	0.03		3.98	0.03		4.61	0.02	
④ 50 or above	57	4.17	0.10		3.89	0.13		4.45	0.09	
**Marital status**				0.93			0.42			1.14
Married	1,258	4.28	0.02		3.95	0.02		4.60	0.02	
Other	192	4.23	0.05		3.92	0.06		4.55	0.05	
**Education Level**				37.29 ***			31.62 ***			28.89 ***
① Junior high or under	162	3.95	0.06		3.53	0.07		4.36	0.06	
② Senior high	601	4.20	0.03		3.88	0.04		4.53	0.03	
③ College or above	690	4.40	0.02	③ > ② > ①	4.09	0.03	③ > ② > ①	4.70	0.02	③ > ② > ①
**Family type**				3.53 ***			3.16 **			3.27 **
Nuclear family ^c^	856	4.32	0.02		4.00	0.03		4.64	0.02	
Others	606	4.19	0.03		3.86	0.04		4.53	0.03	
**Occupation**				9.17 ***			9.18 ***			5.53 ***
① Agriculture-industry	313	4.13	0.04	① < ③	3.77	0.05	① < ③	4.50	0.03	① < ③
② Business	154	4.21	0.05	② < ③	3.85	0.07	② < ③	4.58	0.05	
③ Service- public, social and health related	332	4.44	0.03		4.16	0.04		4.71	0.03	
④ Service-others	369	4.27	0.03	④ < ③	3.95	0.05	④ < ③	4.60	0.03	
⑤ Unemployed	269	4.26	0.04	⑤ < ③	3.92	0.05	⑤ < ③	4.60	0.04	
**Annual family income**				9.81 ***			6.85 ***			9.28 ***
① 300,0000 or under	292	4.11	0.04		3.77	0.06		4.46	0.04	
② 300,001–600,000	425	4.23	0.03		3.89	0.04		4.57	0.03	
③ 600,001–800,000	262	4.31	0.04	③ > ①	3.99	0.05		4.64	0.03	③ > ①
④ 800,000–1,000,000	180	4.41	0.04	④ > ① & ②	4.10	0.06	④ > ①	4.72	0.04	④ > ①
⑤ 1,000,001 or above	270	4.39	0.04	⑤ > ① & ②	4.06	0.05	⑤ > ①	4.70	0.03	⑤ > ①
**School location**				6.74 ***			6.99 ***			3.95 **
①North	544	4.28	0.03	① > ③	3.94	0.04	① > ③	4.16	0.02	① > ③
②Central	350	4.33	0.03	② > ③	4.04	0.05	② > ③	4.64	0.03	② > ③
③South	258	4.11	0.04		3.73	0.05		4.48	0.04	
④East	314	4.30	0.04	④ > ③	4.00	0.05	④ > ③	4.60	0.04	④ > ③
**Children’s grade**				0.15			0.59			0.14
①Grades 1–2	478	4.26	0.03		3.92	0.04		4.60	0.03	
②Grades 3–4	487	4.28	0.03		3.97	0.04		4.58	0.03	
③Grades 5–6	501	4.26	0.03		3.92	0.04		4.60	0.03	
**Smoking status**				−12.60 ***			−12.27 ***			−9.72 ***
Smoker	336	3.87	0.04		3.43	0.05		4.31	0.03	
Non-smoker	1,105	4.39	0.02		4.10	0.02		4.68	0.02	
**Agree with smoking restrictions in the home**				6.74 ***			6.63 ***			5.40 ***
Yes	1,217	4.32	0.02		4.01	0.02		4.64	0.02	
No	220	3.95	0.05		3.54	0.07		4.35	0.05	

^a^ f value by one-way ANOVA test for comparison of three and more variables, t value by independent t test for comparison of two variables; ^b^ standard deviation; ^c^ living with spouse and children only; * *p* < 0.05, ** *p* < 0.01, *** *p* < 0.001.

**Table 3:** Hierarchical Regression Analysis for factors predicting parents’ evaluation of the consequences of parental smoking in the presence of children.

Independent variables	Model 1		Model 2		Model 3	
	β	*p*	β	*p*	β	*p*
**Current smoker (ref.: non-smoker)**	−0.52	<0.0001	−0.49	<0.0001	−0.42	<0.001
**Agree with having home smoking restrictions (ref.: disagree)**			0.28	<0.0001	0.25	<0.001
**Gender (ref.: male)**					0.07	0.008
**Education level (ref.: junior or under)**						
Senior high					0.19	<0.001
College or above					0.25	<0.001
**Occupation (ref.: Service-public, social and health related)**						
Agriculture-industry related					−0.15	0.004
Business					−0.07	0.23
Service-others					−0.04	0.45
unemployed					−0.08	0.13
**Nuclear family type (ref.: others)**					0.04	0.19
**Annual family income (NTD) (ref.: 300,000 or less)**						
300,001–600,000					0.05	0.35
600,001–800,000					0.05	0.34
800,001–1,000,000					0.11	0.07
1,000,001 or above					0.08	0.20
**Children’s school location (ref.: East)**						
North					−0.01	0.79
Central					0.01	0.82
South					−0.05	0.39
**Adjusted R^2^**	11.21%		13.65%		17.23%	
**Models**			2 *vs.* 1		3 *vs.* 2	
**ΔR^2^**			2.44%		3.58%	
**ΔF**	182.75 ***		88.65 ***		12.37 ***	

* *p <* 0.05, ** *p* < 0.01, *** *p <* 0.001.

**Table 4:** Hierarchical Regression Analysis for factors predicting parental perceptions of parents smoking in the presence of children.

Independent variables	Model 1		Model 2		Mode13		Model 4	
	β	*p*	β	*p*	β	*p*	β	*p*
**Risk awareness of parents smoking in the presence of children**	0.60	<0.0001	0.55	<0.0001	0.55	<0.0001	0.54	<0.001
**Current smoker (ref.: nonsmoker)**			−0.20	<0.0001	−0.18	<0.0001	−0.08	0.04
**Agree with having home smoking restrictions (ref.: disagree)**					0.15	<0.0001	0.16	<0.001
**Gender (ref.: male)**							0.16	<0.001
**Education level (ref.: junior or under)**								
Senior high							0.05	0.27
College or above							0.11	0.03
**Occupation (ref.: Service-public, social and health related)**								
Agriculture-industry related							0.009	0.83
Business							0.01	0.80
Service-others							−0.01	0.77
unemployed							−0.05	0.24
**Nuclear family type (ref.: others)**							0.02	0.55
**Annual family income (NTD) (ref.: 300,000 or less)**								
300,001–600,000							0.04	0.27
600,001–800,000							0.003	0.94
800,001–1,000,000							0.06	0.27
1,000,001 or above							0.11	0.03
**Children’s school location (ref.: East)**								
North							0.02	0.51
Central							−0.03	0.41
South							−0.03	0.51
**Adjusted R^2^**	36.93%		37.68%		39.41%		41.62%	
**Models**			2 *vs.* 1		3 *vs.* 2		4 *vs.* 3	
**ΔR^2^**			0.75%		1.73%		2.21%	
**ΔF**	858.7 ***		54.22 ***		67.39 ***		11.97 ***	

* *p* < 0.05, ** *p* < 0 .01, *** *p* < 0.001.

Parents in this group displayed a greater acceptance of parental smoking in the presence of children and were less concerned with its influence. Other than these sociodemographic factors, parents who were current smokers displayed greater acceptance with parental smoking in the presence of children and agreed less with its adverse consequences. These results indicate that parental smokers, and especially paternal smokers with lower socioeconomic status, must be considered because evidence has shown a higher level of children’s exposure to SHS when parents display more positive attitude toward it or less risk awareness of its harmful effects [17]. In Taiwan, the percentage of male smokers is substantially higher than that of female smokers: 45%–50% of men between 30 and 50 years of age smoke, compared with only 5%–8% of women in the same age group [30]. However, previous intervention programs emphasizing the harmful effects of SHS have targeted mothers [31,32,33,34,35,36,37], who either smoked or did not smoke, rather than fathers. The results of this study suggest that health care providers might need to target paternal smokers in delivering health education regarding the harmful effects of SHS. Health education efforts would also be more cost-effective if they were targeted at people lacking information, because the majority of people are aware of the harmful effects of SHS [7,8]. The government and health care delivery systems need to establish an effective approach to convey information to this specific group.

Parents who agreed with having home smoking restrictions perceived parental smoking more negatively and were more in agreement regarding its adverse consequences. Especially parents’ evaluation of the consequence of parental smoking in the presence of their children predicted their perceptions of this behavior. These findings indicated that promoting parents’ acceptance of establishing rules for a smoke-free home, as well as providing health education to enhance awareness of the risk of SHS, may improve parental attitudes against parental smoking. Parents present more effort to prevent children’s exposure to SHS at home if they express more attitudes against it [38,39]. Therefore, we suggest that a more effective approach to promote parents’ preventive efforts would be by both improving parents’ awareness of the harm caused by parents smoking and reinforcing negative attitudes toward parental smoking rather than simply improving knowledge. A previous study found the similar results [40].

The parents in this study exhibited negative perceptions and evaluations of the negative consequences of exposing children to parental smoking. However, compared with other PSPC subscales, the “rational need” subscale demonstrated a fairly neutral perspective (mean score 2.41), indicating that parents did not completely deny the rational needs of smokers. These included the need to smoke and the cultural context of smoking. Previous studies [20,39] have shown that the social/cultural context often results in the failure of parents to prevent SHS at home. To prevent parental smoking in the presence of children, health educators should consider interventions aimed at increasing the awareness of nonsmokers, especially children, and their need for a smoke-free environment and reforming social customs related to smoking.

Over-fitting might have occurred during the process of model building in regression analysis, leading to a poor model. We suggest the use of assessment techniques, such as cross-validation from data of a larger sample, to detect this problem in a further study. One limitations of this study is the use of a convenience sample for parents of school-aged children in Taiwan, which limits the generalizability of the results. However, we selected study participants from four locations across the nation (north, central, south, and east). This wide range of sampling should provide a greater variety of parental characteristics, thus increasing the representativeness of the results. Nevertheless, random sampling and the inclusion of parents with younger children from more geographic locations is suggested for further study to increase the representativeness and generalizability of the results. Another limitation is the use of a cross-sectional study design, which makes it difficult to identify causal relationships. We suggest a longitudinal study design in the future to determine causality.

## 5. Conclusions

This study examined factors associated with parents’ perceptions about parental smoking in the presence of children, and their evaluation of its consequences. We identified various sociodemopraphic characteristics, such as gender, education level, and occupation type/annual family income, as predictors of perceptions and evaluation of consequence. Parents’ smoking status, agreement with home smoking bans, and certain demographic characteristics were associated with perceptions of parental smoking and its consequences. Parents’ evaluation of the consequences of parental smoking in the presence of children also affected their perception toward parental smoking. Identifying parents with specific characteristics is critical to effectively using resources to target specific groups with health education. We suggest implementing rules for a smoke-free home, providing health education and reinforcing negative attitudes toward parental smoking can raise parents’ effort to prevent children from SHS exposure caused by parental smoking. 

## Supplementary Material

**Scale of “Perceptions of parents smoking in the presence of children” (PSPC)**

**Table:** **Q: How do you perceive parents’ smoking in the presence of their children?** 5 ➔ “strongly agree”, 4 ➔ “agree”, 3 ➔ “no comment”, 2 ➔ “disagree”, 1 ➔ “strongly disagree”.

	Item content	Level of agreement				
		5	4	3	2	1
1	I think media reports claiming “ETS is harmful to health” are overstated	□	□	□	□	□
2	Only smoke from tobacco pipes impact children’s health	□	□	□	□	□
3	I think only ill children are affected by secondhand smoke	□	□	□	□	□
4	Smoking is a negative role model	□	□	□	□	□
5	Smoking in the presence of children makes me feel guilty	□	□	□	□	□
6	Smoking has a bad impact on children’s health	□	□	□	□	□
7	Smokers’ smoking needs should be respected	□	□	□	□	□
8	Smoking is part of the cultural	□	□	□	□	□

**Scale of “Consequence Evaluation of Parents Smoking in the Presence of Children” (CESPC)**

**Table:** **Q: How do you evaluate the consequence of parents smoking in the presence of children?** 5 ➔ “strongly agree”, 4 ➔ “agree”, 3 ➔ “no comment”, 2 ➔ “disagree”, 1 ➔ “strongly disagree”.

	Item content	Level of agreement				
		5	4	3	2	1
1	Children’s health is affected	□	□	□	□	□
2	Air quality within the home is affected	□	□	□	□	□
3	Clothes and furniture smell of tobacco smell	□	□	□	□	□
4	Children will imitate parents’ smoking behavior	□	□	□	□	□
5	Children’s emotional state is affected	□	□	□	□	□
6	Children’s interpersonal relationships are affected	□	□	□	□	□
